# Threats of Pollutants Derived from Electronic Waste to Marine Bivalves: The Case of the Rare‐Earth Element Yttrium

**DOI:** 10.1002/etc.5508

**Published:** 2022-12-13

**Authors:** Madalena Andrade, Amadeu M. V. M. Soares, Montserrat Solé, Eduarda Pereira, Rosa Freitas

**Affiliations:** ^1^ Departamento de Biologia & CESAM Universidade de Aveiro Aveiro Portugal; ^2^ Departamento de Recursos Marinos Renovables Instituto de Ciencias del Mar ICM‐CSIC Barcelona Spain; ^3^ Departamento de Química & CESAM/LAQV‐REQUIMTE Universidade de Aveiro Aveiro Portugal

**Keywords:** Bioconcentration, cellular damage, metabolism, mussels, oxidative stress, rare‐earth elements

## Abstract

The production of electrical and electronic equipment waste (e‐waste) is increasing at an alarming rate worldwide. This may eventually lead to its accumulation in aquatic environments, mainly because of the presence of nonbiodegradable components. The rare‐earth element yttrium (Y) is particularly relevant because it is present in a wide variety of electro‐based equipment. Within this context, the present study investigated the biological consequences of anthropogenic Y exposure in *Mytilus galloprovincialis*. Mussels were exposed to Y (0, 5, 10, 20, 40 μg/L) for 28 days, and their bioaccumulation and biomarkers related to metabolism, oxidative stress defenses, cellular damage, and neurotoxicity were evaluated. The results revealed that tissue Y content increased at increasing exposure concentrations (though the bioconcentration factor decreased). At the lowest Y dosage (5 µg/L), mussels lowered their electron transport system (ETS) activity, consumed more energy reserves (glycogen), and activated superoxide dismutase activity, thus preventing cellular damage. At the highest Y dosage (40 μg/L), mussels reduced their biotransformation activities with no signs of cellular damage, which may be associated with the low toxicity of Y and the lower/maintenance of ETS activity. Although only minor effects were observed, the present findings raise an environmental concern for aquatic systems where anthropogenic Y concentrations are generally low but still may compromise organisms' biochemical performance. Particularly relevant are the alterations in energy metabolism and detoxification processes for their longer‐term impacts on growth and reproduction but also as defense mechanisms against other stressors. *Environ Toxicol Chem* 2023;42:166–177. © 2022 The Authors. *Environmental Toxicology and Chemistry* published by Wiley Periodicals LLC on behalf of SETAC.

## Graphical abstract

Biomarkers altered with different yttrium concentrations. ETS = electron transport system; GLY = glycogen; SOD = superoxide dismutase; GR = glutathione reductase; GSTs = glutathione *S*‐transferases; CbEs = carboxylesterases; LPO = lipid peroxidation.

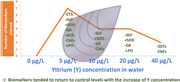

## INTRODUCTION

The manufacturing sector of electric and electronic equipment (EEE) is quickly expanding in tandem with industrialization, economic and technological development, as well as luxurious lifestyles (Ahirwar & Tripathi, 2021). Consequently, an alarming growth rate of EEE waste (e‐waste) of 3%–4% per year is anticipated, with an expectation of 74 million metric tons by 2030 (Forti et al., 2020). Thus, e‐waste is becoming a global problem not only because of its vast volume but also because of its associated environmental risks. This is because most components of e‐waste are nonbiodegradable, potentially may accumulate in the environment, and therefore place biological resources and human health at risk (Wang et al., 2016; World Economic Forum, 2019). Some of the most essential components of EEE are the rare‐earth elements (REEs), thanks to their desirable physical and chemical characteristics, such as high thermal stability and electrical conductivity, strong magnetism, and high luster, besides other optical properties, such as fluorescence and ability to emit light in the visible range (Dushyantha et al., 2020). As a consequence of their wide and increasing use, REEs have been identified in several environmental compartments (see Kulaksız & Bau, 2011; Li et al., 2013; Lu et al., 2003; Protano & Riccobono, 2002), with numerous reports confirming their presence in marine biota (Gwenzi et al., 2018; Lortholarie et al., 2020; Wang et al., 2019).

Yttrium (Y) is an element included in the REE lanthanides group because of its shared chemical characteristics of a trivalent cation (Dinér, 2016). This element has a wide range of uses in industry, including alloys, phosphors for lamps and displays, and microwave, radar, laser, and optical applications (Voncken, 2016; Zhang et al., 2017). In fact, because of its ample use, Y has been found in natural water compartments including rivers at concentrations ranging from 0.05 to 98 μg/L in Europe, from 0.08 to 0.46 μg/L in Africa, from 0.03 to 1.00 μg/L in North America, and from 0.001 to 45 μg/L in Asia (Gaillardet et al., 2003; Reimann & Caritat, 1998). In seawater, Y has been detected at ranges from 0.02 to 0.04 μg/L in the Mediterranean Sea and from 0.005 to 0.026 μg/L in the western South Pacific Ocean (East Caroline, Coral Sea, and South Fiji basins; Bau et al., 1997; Zhang & Nozaki, 1996).

Despite Y's recognized occurrence in aquatic environments, its impact on aquatic organisms is still poorly understood, with limited studies addressing its toxicological effects. In an in vivo study with rainbow trout (*Oncorhynchus mykiss*), Y affected the transcription of genes involved in protein denaturation at concentrations approximately 120 times lower than the 96‐h median lethal concentration of 0.7 mg/L (Dubé et al., 2019). In an in vitro study with hepatocytes of the same fish species exposed to Y for 48 h at 15 °C, increases in glutathione *S*‐transferases (GSTs) activity were seen at >0.5 mg/L and in arachidonate cyclooxygenase activity and metallothionein (MT) content at >2.5 mg/L (Hanana et al., 2021). In the freshwater mussel *Dreissena polymorpha* after 28 days of exposure to Y (10, 50, 250, and 1250 μg/L) anti‐inflammatory and genotoxic effects, as well as changes in the gene expression of catalase (CAT), GSTs, and cytochrome c oxidase 1, occurred (Hanana et al., 2018). In the marine oyster *Crassostrea gigas* embryos, median effective concentrations of 147 μg/L (24 h) and 221.9 μg/L (48 h) were estimated (Moreira et al., 2020). Given the economic and ecological significance of marine and estuarine ecosystems, the lack of information on the impacts of Y on their inhabiting species is a cause of concern, particularly when considering long‐term and environmentally realistic exposures. With the present analytical developments, it is possible to determine, and therefore predict, the consequences of low‐exposure concentrations for the majority of chemicals of concern. This improves the effectiveness of assays that mimic environmentally relevant concentrations and provides formerly lacking data on these emerging chemicals to safety regulators (Weltje & Sumpter, 2017).

Information on an organism's physiological status can be gathered from its metabolic performance and usage of energy reserves (De Coen & Janssen, 2003), which, consequently, can inform about their health status alterations when subjected to stressful conditions. Further information, however, can also be obtained at the cellular level because organisms under stress conditions may generate an excess of reactive oxygen species (ROS), which, if not efficiently eliminated by antioxidant mechanisms, may cause cellular damage (Catalá, 2009; Regoli & Giuliani, 2014). Moreover, organisms use Phase I and/or Phase II biotransformation enzymes to catalyze the conversion of a wide range of organic contaminants and facilitate their excretion from cells (Regoli & Giuliani, 2014; Yan, 2014). However, these detoxification mechanisms can also be affected by metal contamination (Dobritzsch et al., 2020; Hauser‐Davis et al., 2021). Beyond these impacts generated on metabolism and oxidative status, neurotransmission impairment can also be assessed through the inhibition of acetylcholinesterase (AChE) activity, especially by organophosphorus pesticide exposures (English & Webster, 2012). However, metals and nanoparticles have also been responsible for AChE inhibition in marine invertebrates (Brown et al., 2004; De Marchi et al., 2018; Perić et al., 2017). Assessment of biomarkers related to these former mechanisms has been revealed to be useful for evaluating the consequences of anthropogenic and natural factors (Lomartire et al., 2021). However, a number of limitations frequently arise, such as those related to data acquisition; and, in this case, the use of multivariate methods in statistical analysis is encouraged (Beliaeff & Burgeot, 2002). Biomarker response indexes, such as the integrated biomarker response (IBR), may combine the overall effects of multiple stressors and facilitate data interpretation.

In light of the aforementioned concerns, the present study evaluated the impact of Y on the mussel *Mytilus galloprovincialis*’ biochemical and physiological performance considering several endpoints. To achieve this, mussels were subjected to a range of environmentally relevant concentrations of Y (0, 5, 10, 20, and 40 μg/L) for 28 days. Besides Y concentration in mussel tissues, several biomarkers related to the changes in their metabolic capacity, energy reserves, oxidative status, and neurotoxicity were assessed.

## METHODOLOGY

### Sampling and experimental conditions

Adult *M. galloprovincialis* of similar size (length 59.1 ± 2.9 mm, width 33.0 ± 2.0 mm) were gathered during low tide in July 2020 from the Ria de Aveiro (northwest of Portugal). Organisms were transferred to the laboratory and allowed to acclimate for 14 days in synthetic seawater (made with deionized water and Tropic Marin® Sea Salt), which was replaced twice in the first and once in the second week. During these 14 days, organisms were maintained under constant aeration at control conditions (temperature 17.0 ± 1.0 °C, pH 8.0 ± 0.1, salinity 30 ± 1, and natural photoperiod) which mimic those at the sampling location. Soon after the initial 5 days of acclimation, organisms were fed every other day with a concentration of 150 000 cells/mussel/day of AlgaMac Protein Plus (Aquafauna BioMarine®). This is a freeze‐dried mix constituted of 39% protein, 20.4% lipid, and 20.6% carbohydrates, with several heterotrophic and phototrophic species, vitamins, attractants, and pigmentation.

After the 14 acclimation days, mussels were distributed into different aquaria (five individuals per aquarium and three aquaria per treatment) each containing 3 L of synthetic seawater. Two more aquaria per treatment under the same conditions but without mussels (positive controls) were also considered. Ambient conditions were maintained, similar to those of the acclimation period, during the experimental time of 28 days with different treatments: 0, 5, 10, 20, and 40 μg/L of Y. The Y concentrations used were selected considering values reported from pristine to contaminated aquatic systems (Bau et al., 1997; Gaillardet et al., 2003; Reimann & Caritat, 1998; Zhang & Nozaki, 1996).

A stock solution of 10 mg/L of Y was obtained by diluting a commercial solution (1000 mg/L; Inorganic Ventures) in ultrapure water. During the experimental period, mussels were fed three times per week with the same concentration of Algamac Protein Plus described previously, and seawater was renewed once a week, reestablishing the desired Y concentration and pH/salinity conditions.

For Y quantification, seawater samples were collected from all aquaria and two positive controls per treatment right after dosing, to assess the real Y concentrations during the first two weeks. To verify the stability of the selected Y concentrations, seawater samples were also collected from positive controls right before water renewal.

Organisms were sampled at the end of the exposure period (28 days), promptly frozen in liquid nitrogen, and kept at −80 °C until analysis. Three mussels per aquarium (nine per treatment) were considered for biochemical measures, and one of them (three per treatment) was used for Y quantification. The whole soft tissue of each mussel was manually homogenized using a pestle and mortar, with liquid nitrogen. Each mussel was divided into five aliquots of 0.5 g each and kept at −80 °C until Y quantification and biochemical determinations.

### Y quantification in seawater and mussel soft tissue

Yttrium was quantified using inductively coupled plasma mass spectroscopy (ICP‐MS) on a Thermo ICP‐MS XSeries paired with a Burgener nebulizer after water sample acidification with HNO_3_ to pH <2. The quantification limit for water samples was considered to be the lowest standard (0.02 µg/L). After prior microwave‐assisted acid digestion, ICP‐MS was also used to determine the total Y content of the organisms' tissue. In Teflon vessels, 200 mg of each freeze‐dried sample were weighed separately and digested with 1 mL of HNO_3_ 65% (v/v), 2 mL of H_2_O_2_, and 1 mL of ultrapure H_2_O thereafter. Afterward, the Teflon vessels were placed in a CEM MARS 5 microwave for 15 min to an increasing temperature up to 170 °C and then remained at this temperature for another 5 min. After allowing the vessels to cool, samples were placed in polyethylene flasks that were then filled with ultrapure water to a final amount of 25 mL at room temperature until Y determination. Digested blanks (microwave vessels without samples), duplicates, and the certified reference material BCR‐668 (mussel tissue; 59 ± 5 μg/kg of Y) were all considered for quality control. The concentration of Y in digested blanks was below the quantification limit of the methodology (0.02 µg/L; lowest standard concentration employed in the calibration curve), whereas recovery values from the certified reference material (three replicates) ranged from 90% to 91%, validating the digestion and quantification protocols.

The concentration of Y in water samples from the control (0 µg/L) treatment was consistently below the quantification limit (0.02 µg/L), and values obtained in seawater after dosing can be seen in Table 1. The levels of Y in the seawater measured from positive controls immediately after dosing and before water renewal as well as corresponding coefficients of variation are presented also in Table 1. Measured and nominal concentrations in seawater coincided, and the coefficients of variation for each treatment were <20%, confirming the stability of Y concentrations in water during 2 weeks of exposure. The concentration of Y quantified in the mussels' tissue for each treatment can be seen in Table 2.

**Table 1:** Yttrium concentration in water samples during 2 weeks of exposure

	Aquaria with mussels	Aquaria without mussels (positive controls)		
Nominal yttrium (μg/L)	Real yttrium (µg/L)	Positive controls after dosing	Positive controls after 1 week	Coefficient of variation (%)
0	–	–	–	–
5	6.8 ± 0.7	6.5 ± 0.6	5.3 ± 0.4	18.6
10	10.3 ± 0.9	10.1 ± 0.3	10.2 ± 1.6	1.0
20	20.5 ± 1.8	21.3 ± 1.4	20.5 ± 1.9	3.8
40	43.1 ± 5.3	46.7 ± 4.6	41.5 ± 3.4	11.1

Values are means with standard deviations (±) of (*n* = 4) measures.

**Table 2:** Yttrium concentration in mussel soft tissues and bioconcentration factor after 28 days of exposure to spiked seawater

Y in water (μg/L)	Y in mussel tissue (µg/g)	BCF (L/kg)
0	0.07 ± 0.01 a	–
5	0.21 ± 0.06 b	37 ± 12 A,B
10	0.32 ± 0.07 b,c	33 ± 7 A
20	0.40 ± 0.11 c	20 ± 4 B,C
40	0.64 ± 0.03 d	15 ± 1 C

Values are means with standard deviations (±) of (*n* = 3) measures. Different lowecased letters for Y in mussel tissue or uppercased letters for BCF represent significant differences among tested concentrations in the respective columns.

Y = yttrium; BCF = bioconcentration factor.

### Biological responses: Biochemical parameters

Several biochemical endpoints were assessed: (1) energy metabolism, measured by electron transport system (ETS) activity, glycogen (GLY) and total protein (PROT) contents; (2) antioxidant defenses, measured by superoxide dismutase (SOD), CAT, and glutathione reductase (GR) activities; (3) biotransformation capacity, measured by GSTs and carboxylesterases (CbEs) activities; (4) cellular damage, assessed by lipid peroxidation (LPO) and protein carbonylation (PC) levels; and (5) neurotoxicity, evaluated as AChE inhibition. A TissueLyzer II (Qiagen) set at a frequency of 20 hertz for 90 s was used for whole‐tissue disruption of biological samples, and samples were further homogenized in the appropriate buffer for each assay at a ratio of 1:2 (w/v) and centrifuged at 4 °C afterward. The supernatant was immediately collected and kept at −80 °C if it was not used immediately. For most measures, a potassium phosphate buffer—50 mmol/L at pH 7.0 with 1 mmol/L ethylenediaminetetraacetic acid, 1% (v/v) Triton X‐100, and 1 mmol/L dithiothreitol—was used for sample extraction. For the particular case of LPO measures, the extraction buffer was 20% (w/v) trichloroacetic acid (TCA), and for ETS quantification it was 0.1 mol/L Tris‐HCl buffer at pH 8.5 with 15% (w/v) polyvinylpyrrolidone, 153 µmol/L magnesium sulfate, and 0.2% (v/v) Triton X‐100. Potassium phosphate buffer and TCA extracted samples were centrifuged at 10 000 *g* for 20 min, whereas those extracted with Tris‐HCl buffer were centrifuged at 3000 *g* for the same time period. The methodology used in each biochemical assay was performed as described in Andrade et al. (2022).

### Data analyses

#### Bioconcentration factor

Yttrium was administered directly in the water, rather than in the food; thus, Y bioaccumulation was estimated using the bioconcentration factor (BCF). How much of the Y may have become associated with the freeze‐dried algae and therefore taken up as feed was not calculated. Values of BCF were calculated using the Arnot and Gobas (2006) equation, which is characterized by the ratio of total Y concentration in the organisms’ tissue in regard to total Y concentration in the water compartment.

### Statistical analyses

The biochemical data for all treatments (*n* = 3 per treatment, where one experimental unit was considered to be an aquarium) were subjected to hypothesis testing using nonparametric permutational analysis of variance (PERMANOVA) with one‐factor design (Y concentration) through the PERMANOVA add‐on in PRIMER, Ver 6 (Anderson et al., 2008). Accordingly, the Euclidean distance data matrix from each parameter was individually analyzed using unrestricted permutation of raw data (9999 permutations) and of Type III sums of squares calculation. The null hypothesis of no significant differences existing between mussels exposed to different concentrations was tested. Results obtained from the main and pairwise tests including degrees of freedom, *p* values, and pseudo‐*F* values can be seen in Supporting Information, Tables S1 and S2. The significance threshold was set at *p* < 0.05, and significant differences are characterized by different letters in the figures.

### Integrated biomarker response index

The IBR index Ver 2 (IBRvs2), proposed by Beliaeff and Burgeot (2002) and further modified by Sanchez et al. (2013), was used to integrate the data obtained from the different biochemical parameters and to illustrate the overall biochemical responses in mussels to different Y treatments. This was accomplished by comparing the deviation between biochemical parameter responses from each Y treatment (5, 10, 20, and 40 µg/L) to those measured in the control (0 µg/L). To calculate the IBR index, a log transformation (*Y*_*i*_) was first applied to reduce variance, where *Y*_*i*_ = log (*X*_*i*_/*X*_0_), in which *X*_*i*_ corresponds to individual biomarker data of each Y treatment and *X*_0_ represents the mean reference data from the control treatment. Afterward, *Y*_*i*_ was standardized following *Z*_*i*_ = (*Y*_*i*_ – *μ*)/*σ*, where the general mean (*μ*) and standard deviation (*σ*) of *Y*_*i*_were considered. The biomarker deviation index (*A* = *Z*_*i*_ – *Z*_0_) was then calculated to create a basal line centered on 0 and to represent the variations of the parameters according to this line. Finally, IBRvs2 was calculated as IBRvs2 = Σ|*A*|.

**Figure 1:**
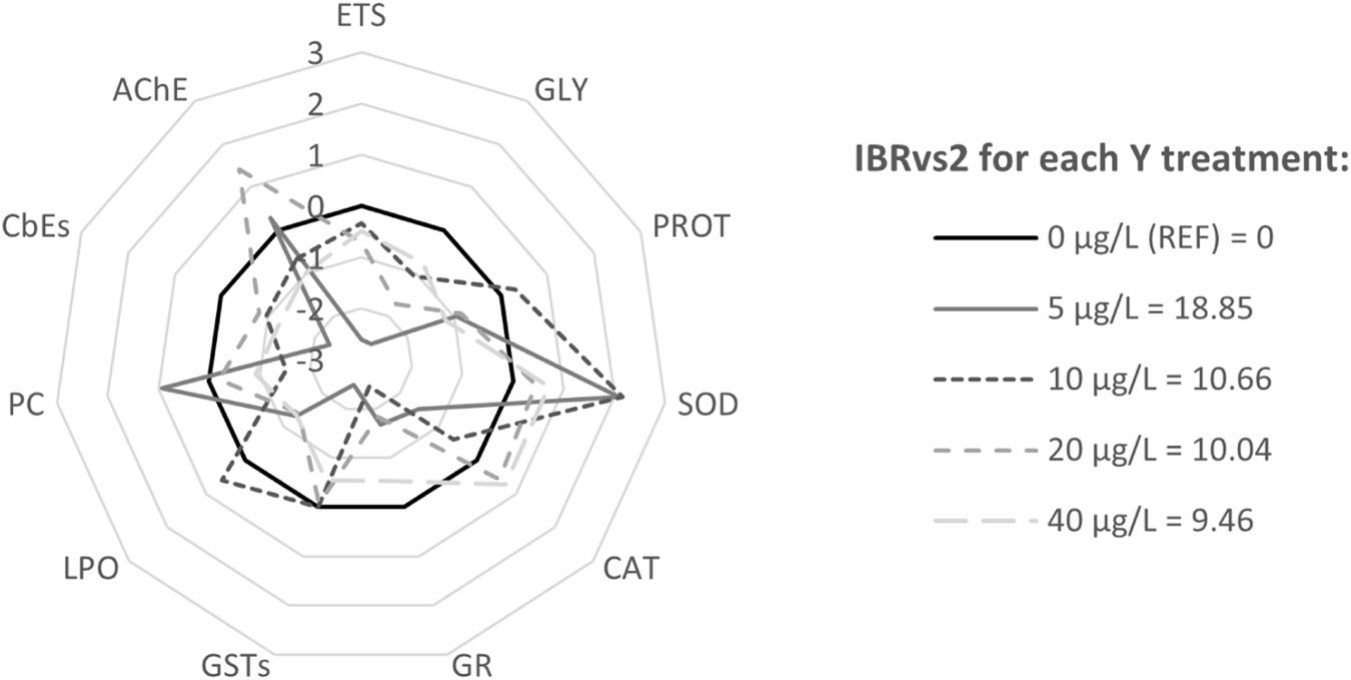
Integrated biomarker response index considering all biochemical parameters used on *Mytilus galloprovincialis* after 28‐day exposure to yttrium at different concentrations: 0 (reference), 5, 10, 20, and 40 μg/L. ETS = electron transport system; AChE = acetylcholinesterase; GLY = glycogen; CbEs = carboxylesterases; PROT = protein; PC = protein carbonylation; SOD = superoxide dismutase; LPO = lipid peroxidation; CAT = catalase; GSTs = glutathione *S*‐transferases; GR = glutathione reductase; IBRvs2 = integrated biomarker response index version 2.

All of the biochemical parameters measured were considered for the IBRvs2 calculations. A star plot was used to display the biomarker deviation index results, with the area outside of the reference line (the line that corresponds to 0 µg/L used as reference data) indicating biomarker induction and the area inside of the reference line, biomarker inhibition. The obtained IBRvs2 values are also indicated in Figure 1. Values were discussed in terms of the overall response provided by the final IBRvs2 model, with higher values corresponding to greater mussel biochemical responsiveness.

## RESULTS

### Y concentration in mussel tissue and BCF

The quantification of Y in mussel tissue confirmed an increasing accumulation trend alongside the exposure gradient, with Y concentrations significantly (*p* < 0.05) lower in control mussels (0 µg/L) but significantly higher in those exposed to 40 µg/L in relation to the intermediate treatments. Of all treatments, only mussels exposed to 10 µg/L showed no significant differences from mussels exposed to 5 and 20 µg/L (Supporting Information, Tables S1 and S2).

The BCF showed a decreasing trend with increasing Y water concentrations. However, no significant differences were reached between BCF values obtained at the lowest (5 µg/L) and the intermediate (10 and 20 µg/L) Y concentrations. Furthermore, BCF values at the highest dosage (40 µg/L) did not show any significant difference from those at 20 µg/L (Supporting Information, Tables S1 and S2).

### Biological responses: Biochemical parameters

#### Metabolic capacity and energy reserves

**Figure 2:**
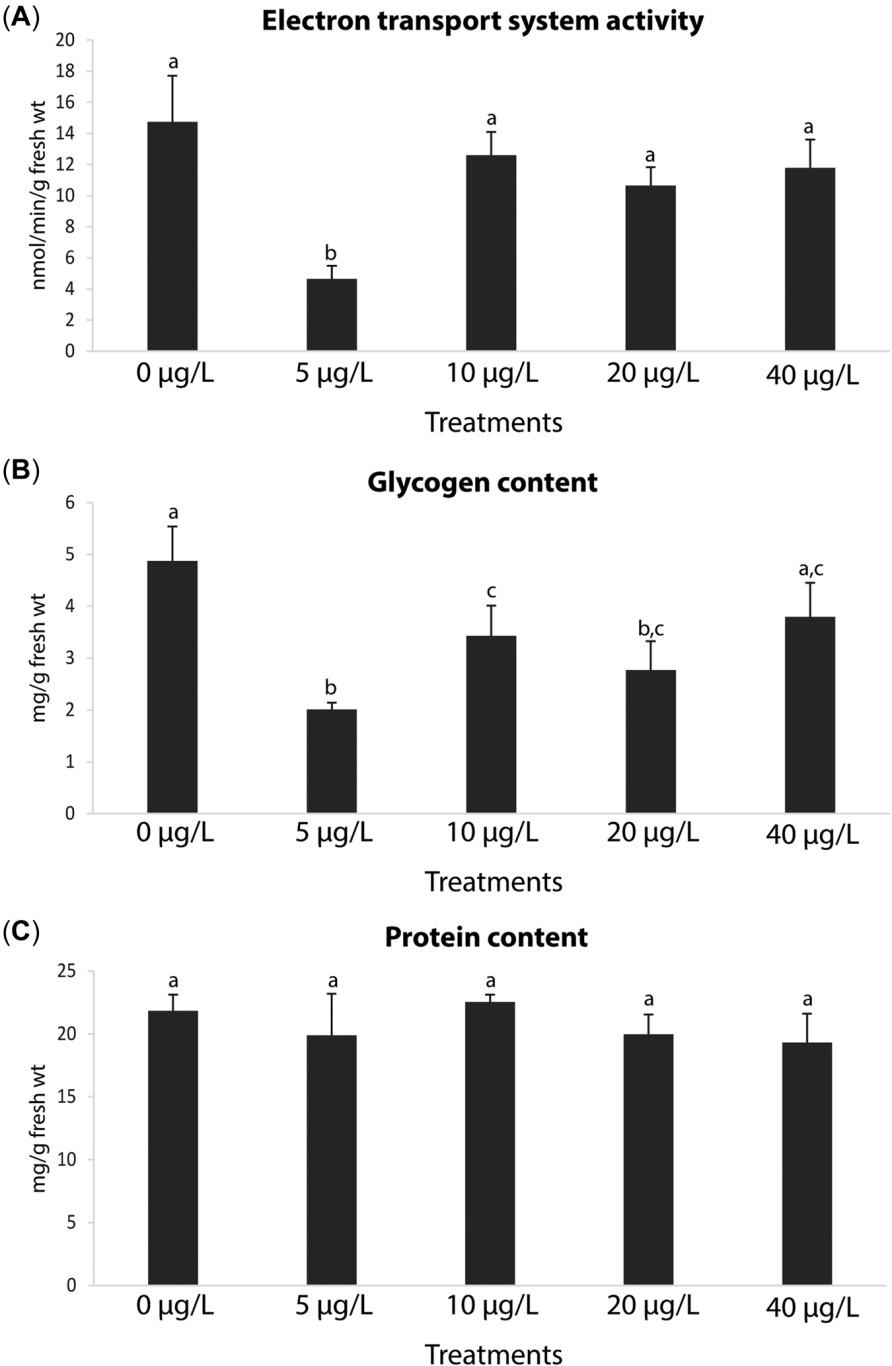
(**A**) Electron transport system activity, (**B**) glycogen content, and (**C**) protein content in *Mytilus galloprovincialis* after 28‐day exposure to yttrium at different concentrations: 0, 5, 10, 20, and 40 μg/L. Results are means with standard deviations. Significant differences (*p* < 0.05) among concentrations are identified with different lowercase letters.

Mussel ETS activity was significantly lower at the lowest Y exposure (5 µg/L), with no significant differences among the remaining treatments (Figure 2A; Supporting Information, Tables S1 and S2). In addition, GLY content was significantly lower at the 5‐, 10‐, and 20‐µg/L treatments, with the lowest values observed at 5 µg/L (Figure 2B; Supporting Information, Tables S1 and S2). By contrast, PROT content did not change significantly among all the treatments (Figure 2C; Supporting Information, Tables S1 and S2).

### Antioxidant and biotransformation enzymes

**Figure 3:**
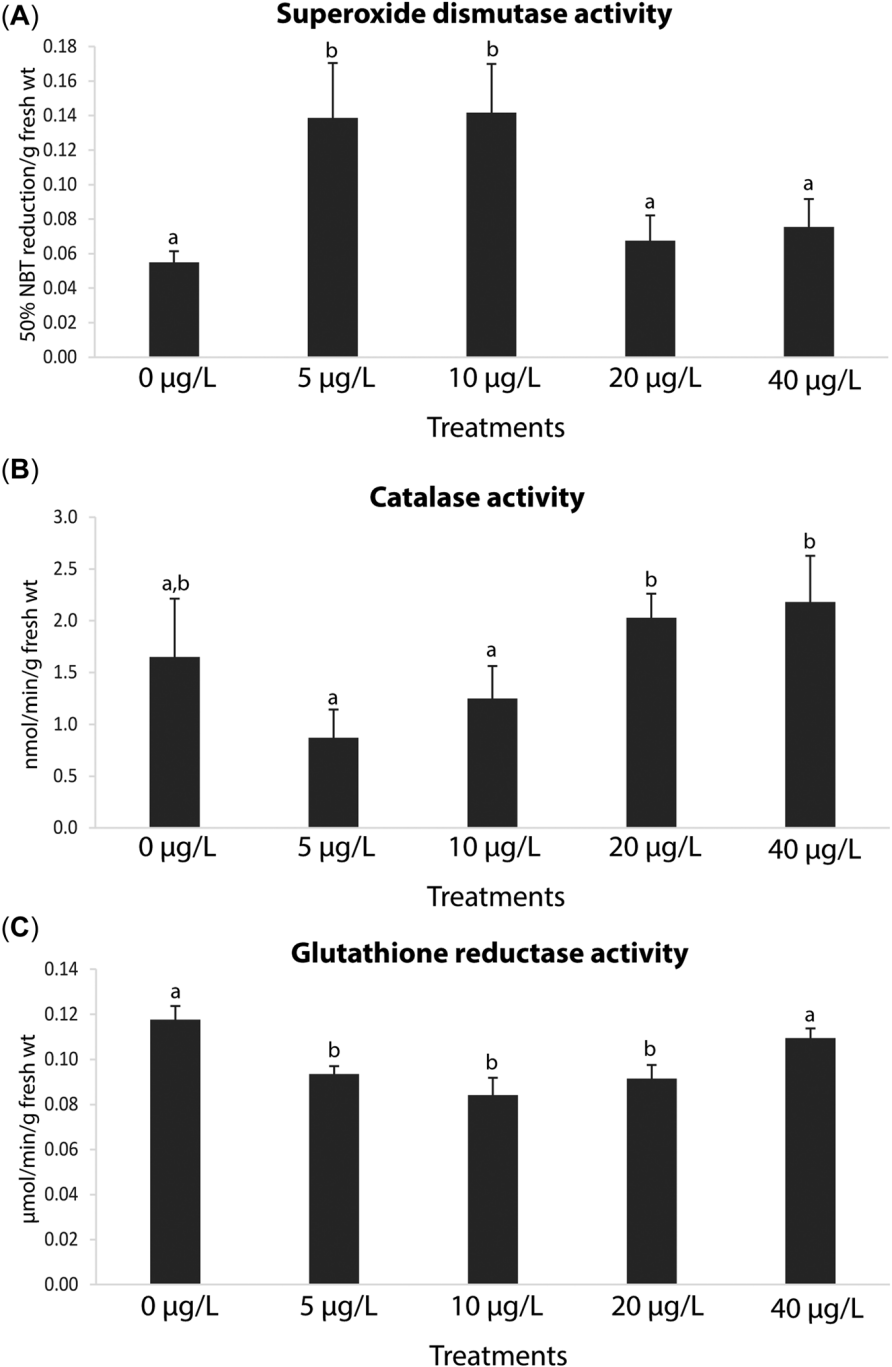
(**A**) Superoxide dismutase activity, (**B**) catalase activity, and (**C**) glutathione reductase activity in *Mytilus galloprovincialis* after 28‐day exposure to yttrium at different concentrations: 0, 5, 10, 20, and 40 μg/L. Results are means with standard deviations. Significant differences (*p* < 0.05) among concentrations are identified with different lowercase letters.

Mussel SOD activity was significantly higher at the 5‐ and 10‐µg/L treatments, with no significant differences between 0 µg/L (control) and the other concentrations (20 and 40 µg/L; Figure 3A; Supporting Information, Tables S1 and S2). Moreover, CAT activity was significantly lower at the 5‐ and 10‐µg/L treatments, in contrast to the other treatments (20 and 40 µg/L; Figure 3B; Supporting Information, Tables S1 and S2), albeit they did not differ significantly from the control. Furthermore, GR activity was significantly lower at the 5‐, 10‐, and 20‐µg/L treatments, with no significant differences between the control and the highest exposure (40 µg/L; Figure 3C; Supporting Information, Tables S1 and S2).

**Figure 4:**
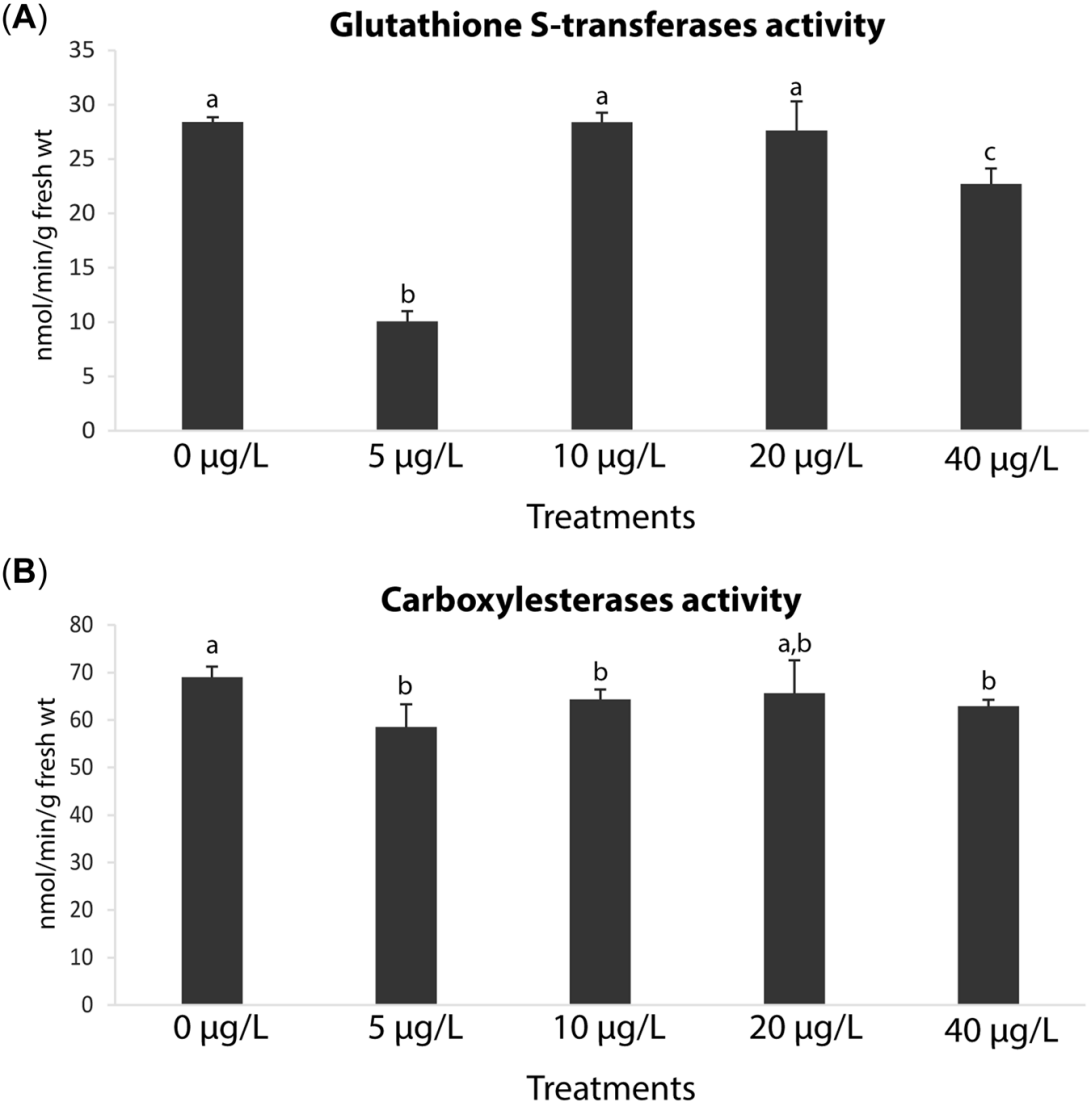
(**A**) Glutathione *S*‐transferases activity, and (**B**) carboxylesterases activity in *Mytilus galloprovincialis* after 28‐day exposure to yttrium at different concentrations: 0, 5, 10, 20, and 40 μg/L. Results are means with standard deviations. Significant differences (*p* < 0.05) among concentrations are identified with different lowercase letters.

Mussel GSTs activity was significantly lower in the 5‐ and 40‐µg/L treatments, in contrast to the remaining ones (0, 10, and 20 µg/L), with the lowest value seen at 5 µg/L (Figure 4A; Supporting Information, Tables S1, S2). In addition, CbEs activity was significantly lower at the 5‐ and 10‐µg/L treatments and higher at 40 µg/L with respect to the control (Figure 4B; Supporting Information, Tables S1 and S2).

### Oxidative damage

**Figure 5:**
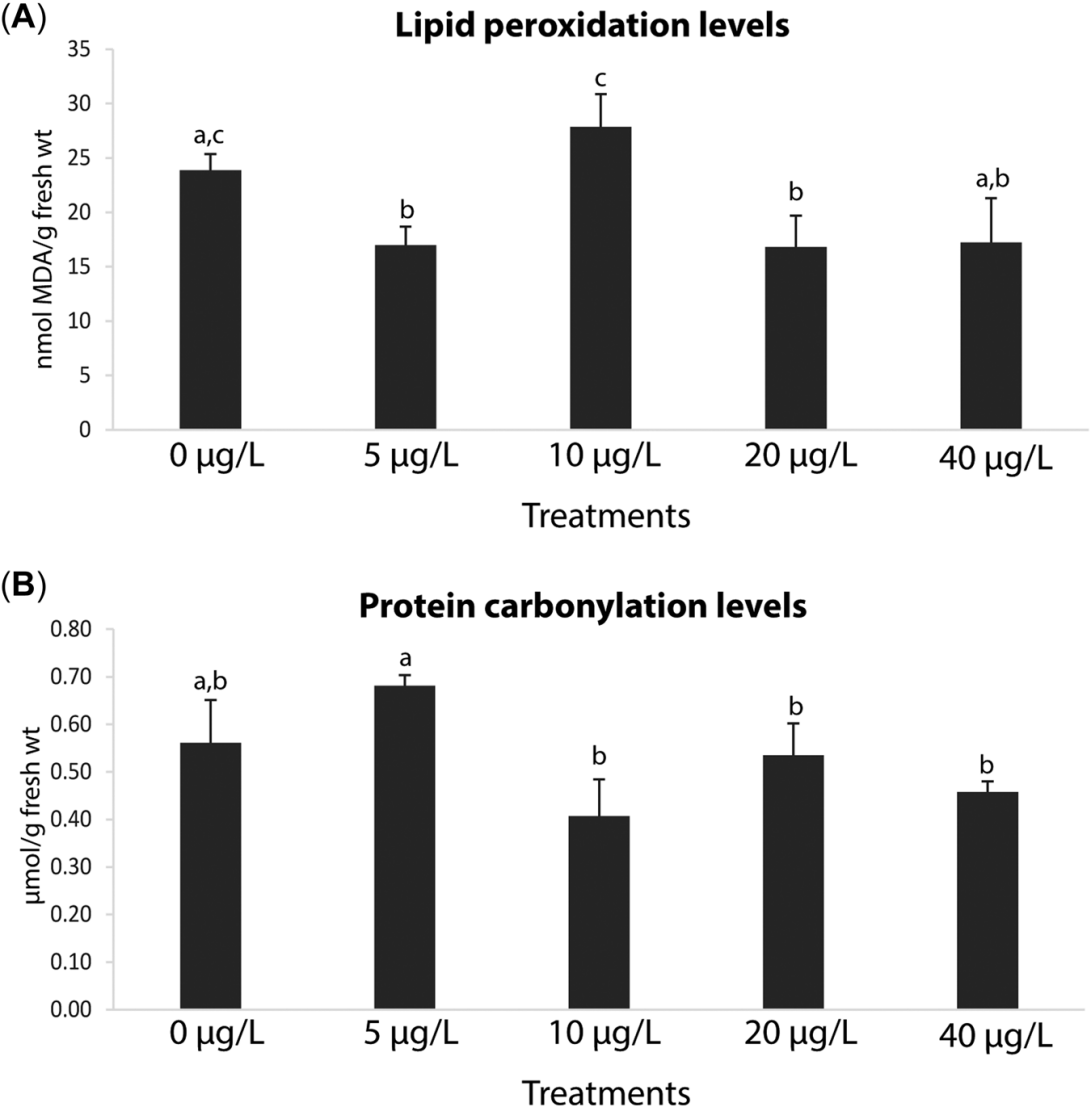
(**A**) Lipid peroxidation levels, and (**B**) protein carbonylation levels in *Mytilus galloprovincialis* after 28‐day exposure to yttrium at different concentrations: 0, 5, 10, 20, and 40 μg/L. Results are means with standard deviations. Significant differences (*p* < 0.05) among concentrations are identified with different lowercase letters. MDA = malondialdehyde.

Mussel LPO levels were significantly lower in the 5‐ and 20‐µg/L treatments with respect to the 10‐µg/L treatment, with no significant differences between these two intermediate treatments (5 and 20 µg/L) and mussels exposed to 40 µg/L or the control (Figure 5A; Supporting Information, Tables S1 and S2). Moreover, PC levels were significantly higher at the lowest Y concentration (5 µg/L), in contrast to the remaining treatments (Figure 5B; Supporting Information, Tables S1 and S2) but not the control.

### Neurotoxicity

**Figure 6:**
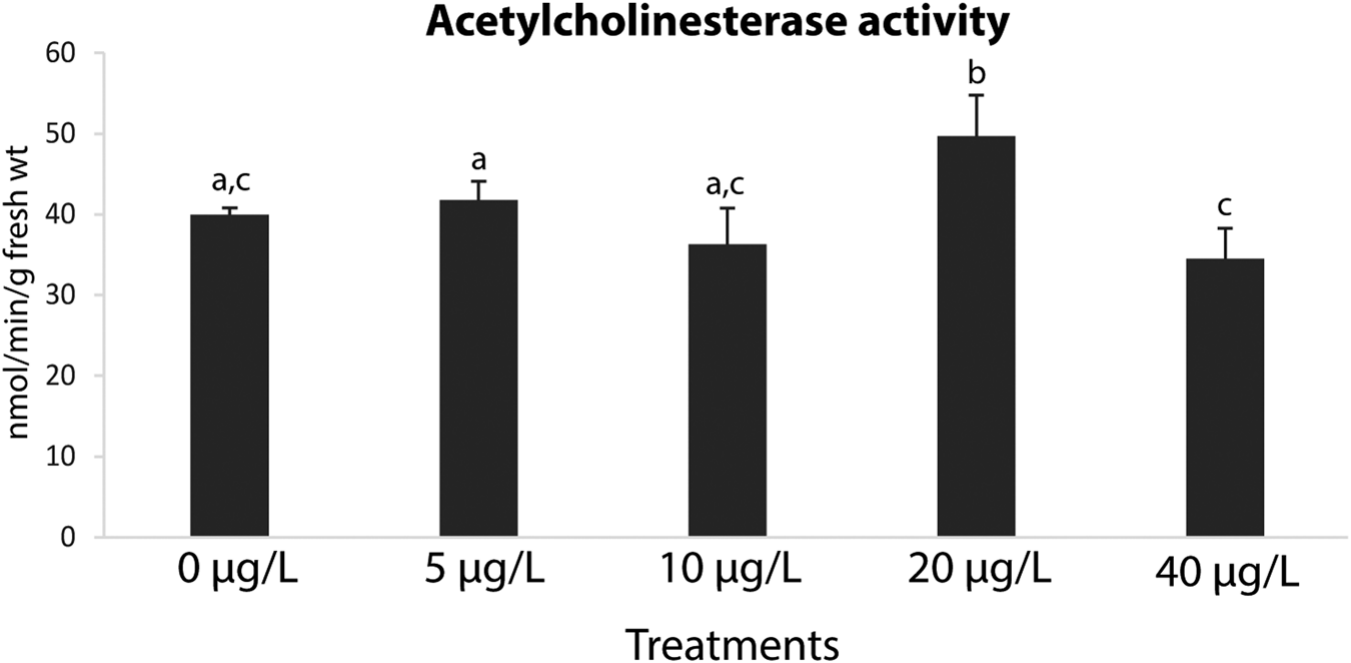
Acetylcholinesterase activity in *Mytilus galloprovincialis* after 28‐day exposure to yttrium at different concentrations: 0, 5, 10, 20, and 40 μg/L. Results are means with standard deviations. Significant differences (*p* < 0.05) among concentrations are identified with different lowercase letters.

Mussel AChE activity was significantly higher only in the 20‐µg/L treatment in comparison with the other treatments (Figure 6; Supporting Information, Tables S1 and S2).

### Integrated biomarker response index

The highest values reported by the IBRvs2 model were those seen at the 5‐µg/L dosage, indicating a greater impact in organisms subjected to this Y treatment. The star plot clearly showed that most biochemical parameter values were lower at this treatment, with the exception of SOD, PC, and AChE, which were higher, contributing to this high score (Figure 1). The results also showed that organisms exposed to the other Y treatments scored similar IBRvs2 values. As determining factors, variations in SOD and GR were primarily responsible for the score in the 10‐µg/L treatment, whereas variations in AChE and GR accounted for those at 20 µg/L. The biomarker variations experienced at the 40‐µg/L treatment were noticeably lower, with CbE, LPO, and PROT modifications being the highest contributors to the IBRvs2 score (Figure 1).

## DISCUSSION

### Bioconcentration of Y in mussels

The present study confirmed that higher Y exposure concentrations resulted in a greater accumulation of Y in mussel tissue. However, the BCF diminished at increasing Y water concentrations, indicating that the mussels tried to limit the accumulation of this element. Available studies on Y accumulation have focused on freshwater, rather than on estuarine and marine environments. For instance, previous studies conducted by Hanana et al. (2018) revealed that when the freshwater mussel *D. polymorpha* was exposed to waterborne Y at 10 µg/L, Y reached a concentration of 0.37 µg/g, which is very similar to the 0.32 µg/g reported in the present study for the same water concentration. However, the accumulation registered in the freshwater species was nearly five times higher (3.43 µg/g) at a waterborne Y concentration of 50 µg/L, which contrasts with our results with marine mussels where the 40‐µg/L treatment yielded bioconcentration of 0.64 µg/g, likely due to Y behavior in two media with different ionic strength. Regarding the ability of mussels to restrict the accumulation of Y, the marine species *M. galloprovincialis* has repeatedly been shown to display this ability for other REEs such as lanthanum (La; Pinto et al., 2019), gadolinium (Gd; Henriques et al., 2019), and neodymium (Nd; Freitas, Costa, et al., 2020). In the present study, limited Y accumulation may result from mussels' adaptive physiological responses, such as reducing filtration rate and/or respiration capacity. Although it was not monitored in the present study, it is well known that mussels may close their valves under unfavorable conditions (Anestis et al., 2007; Gosling, 2003), which would restrain the entrance of Y into the organism. In this situation, biomarkers related to respiratory metabolism could suggest the use of these adaptive responses, which would imply a change from aerobic to anaerobic metabolism. Bivalves are also recognized to sequester foreign chemicals into their shells (see Akagi & Edanami, 2017; Merschel & Bau, 2015; Valdés‐Vilchis et al., 2021), and proteins, such as MTs, are particularly related to metal binding and sequestration. Although MTs are not so investigated in aquatic organisms in terms of REE exposures, MT levels were modulated by Gd in the freshwater mussel *D. polymorpha* (Hanana et al., 2017) and increased in rainbow trout hepatocytes exposed to several REEs including Y (Hanana et al., 2021). Because Y content was measured in the soft part of the mussels, the first hypothesis on metal storage in the shell could not be confirmed. As for the role of MTs, it cannot justify the decrease in BCF but it may account for the lack of biochemical effects at higher Y concentrations in the present study.

### Metabolic capacity and energy reserves

In terms of metabolic capacity, organisms under Y exposure either reduced ETS activity (5 µg/L) or maintained it (10–40 µg/L). This result may indicate that at low Y concentrations organisms can cope with the situation by reducing their aerobic respiration and, thus, restrain Y accumulation. In fact, besides being used as a marker of metabolic activity in marine macrofauna (Cammen et al., 1990), it has been repeatedly proven that a decrease in ETS activity was associated with a reduction in filtration and/or respiration rate, to prevent/limit chemical accumulation in bivalves (see Almeida et al., 2015). In the present study, at higher Y concentrations, ETS activity was maintained at levels similar to control. This is also a mechanism to avoid Y accumulation because enhanced aerobic respiration is expected to face energetic needs during stress. However, a sound explanation for the decrease of BCF observed based solely on ETS results can only be speculative; other biochemical and/or physiological processes, not considered in our study, may have contributed to this decrease. Nevertheless, an association between ETS activity and BCF was previously outlined in mussels with respect to other REEs. For instance, Pinto et al. (2019) found that *M. galloprovincialis* experienced a significant drop in the BCF of La as the concentration increased (0.1–10 mg/L), and this drop in BCF was associated with a decrease in ETS activity. Similar observations were registered in the same mussel species exposed to more comparable Gd concentrations (15–120 µg/L) by Henriques et al. (2019).

In terms of energy reserves, in the present study, GLY content was lowered in contaminated organisms, while PROT reserves were maintained. These results suggest that, despite efforts to avoid Y accumulation by lowering or maintaining aerobic respiration, the presence of Y resulted in greater energetic usage, probably associated with demands in antioxidant and detoxification defenses. Likewise, a decline of glycogen was formerly seen in mussels, *M. galloprovincialis*, exposed to other REEs such as Nd and dysprosium (Dy), which was also indicated to provide protection (Freitas, Cardoso, et al., 2020; Freitas, Costa, et al., 2020). In the particular case of intertidal mussels, the decrease in GLY was also associated with its use in anaerobiosis (De Zwaan, 1983; Hochachka & Mustafa, 1972). This would be further supported by the decrease (and/or maintenance) of ETS observed because organisms may not only limit their aerobic respiration but also shift it to an anaerobic pathway. All of these findings may be of physiological relevance because reproduction and growth could be compromised by reduced respiration/filtration rates and enhanced GLY expenditure at long‐term ambient Y exposures.

### Antioxidant and biotransformation enzymes

The fast response of SOD at relevant Y concentrations (5 and 10 µg/L) indicated that mussels were able to activate this first defense line without the need to enhance their metabolic energy supplies. A fast response by SOD to La (0.1 and 1 mg/L) was described by Pinto et al. (2019) in *M. galloprovincialis*, whereas another antioxidant defense (CAT activity) remained constant. Likewise, Freitas, Costa, et al. (2020) reported that mussels exposed to Nd increased SOD activity at the lowest concentration (2.5 µg/L), whereas CAT only responded at the upper ones (5, 10, and 40 µg/L). Similarly, mussels exposed to Dy enhanced SOD activity in the range of 5, 10, 20, and 40 µg/L, whereas CAT was only activated at the two highest exposures (Freitas, Cardoso, et al., 2020). Particularities between these former responses may be associated with specific REE toxicity, but all coincided with the fact that SOD was a fast response. The Y treatments responsible for a significant SOD activity increase were parallel to CAT activity reduction that could reflect higher H_2_O_2_ production, which in turn caused CAT inhibition. In fact, the increase in intracellular ROS due to H_2_O_2_ overproduction was linked to a reduction in CAT expression (Venkatesan et al., 2006). In addition, the decrease or maintenance of ETS activity observed in the present study under Y exposure could have constrained the responses of GR and even CAT, to a certain extent, because energetic (GLY) reserves would be shifted to maintain anaerobic metabolism. However, inhibition of these antioxidant enzymes by an excess of ROS could be the most likely event because SOD was enhanced. Decreased GR defenses were also reported in freshwater mussels, *Unio tumidus* and *Unio pictorum*, transplanted to metal‐polluted sites (Cossu et al., 1997; Guidi et al., 2010).

The coincident responses of both biotransformation enzymes (CbEs and GSTs), with a decrease at the lowest Y dosage (5 µg/L) support their role as Phase I and II steps in detoxification. The positive relationship between the two enzymes in bivalves has been studied by Solé et al. (2020) in polycyclic aromatic hydrocarbon–contaminated environments, and they suggested CbEs as a good indicator of Phase I metabolism in bivalves. In fact, in the present study, these two biomarkers have also shown a significant positive Pearson correlation value (*r* = 0.679, *p* < 0.05), supporting this proposal. There is also a possible link between GSTs and other antioxidant defenses in mussels, at least in the case of 5 µg/L of Y. Under this treatment, the enhanced production of H_2_O_2_ (likely due to the rise in SOD activity and lack of CAT removal action) may have contributed to the prevention of other antioxidant responses, including GSTs, because ROS are known to interact with cytosolic GSTs, leading to their inactivation (Letelier et al., 2010). Morosetti et al. (2020) observed that in *M. galloprovincialis* exposed to mercury (Hg) or a mixture of Hg and nanoparticles of cerium (CeO_2_), the GSTs activity decrease was associated with a lack of GR response.

### Oxidative damage

In terms of oxidative damage, mussels in the 5‐µg/L Y exposure showed the lowest LPO levels, which could be partly due to the efficiency of SOD at preventing cellular damage but also to decreased ETS activity, which would generate less ROS in the mitochondrial electron transport chain (Phaniendra et al., 2015). The study of Pinto et al. (2019) also revealed a decrease in LPO and PC levels in *M. galloprovincialis* subjected to La, which was correlated with an enhancement of antioxidant enzymes, such as SOD and glutathione peroxidase, and lower ETS activity. Nonetheless, the freshwater mussel *D. polymorpha* exposed to Y (10–250 µg/L) exhibited unaltered LPO levels, despite the fact that these organisms manifested anti‐inflammatory and genotoxic responses (Hanana et al., 2018). It is also likely that the freshwater mussels from that study used other not considered mechanisms to avoid damage.

### Neurotoxicity

Instead of AChE inhibition, as a sign of neurotoxic action, only a rise in AChE activity at 20 µg/L of Y was noticeable. Likewise, in a fish study, Figueiredo et al. (2018) observed that the REE La caused enhanced AChE activity in glass eels, *Anguilla anguilla*, which the authors linked to La binding to the acetylcholine receptors, replacing calcium. In fact, Y has also been shown to interfere with calcium‐dependent processes in vertebrates (Shemarova et al., 2014), which could explain the increase of AChE at particular Y concentrations in mussels.

### Integrated biomarker response index

The application of the IBRvs2 model further confirmed that the lowest Y concentration (5 µg/L) had the greatest effect, which is due to its negative impact on most of the biomarkers evaluated (including CbEs and GSTs decreases). A reduced metabolism was related to a switch to anaerobic respiration (lower ETS and GLY), potentially due to valve closure and reduced filtration rate that refrained Y accumulation. At the same time, the increase in SOD activity at this particular concentration prevented LPO. Moreover, a decrease in the IBRvs2 values (despite an increase in Y water concentrations) was seen, which was in line with less marked biomarker responses and a tendency to return to control values. This trend may be due to the use of other physiological adaptive responses which were not addressed in the present study. Furthermore, because most oxidative stress and neurotoxicity indicators were not affected in the range of selected Y concentrations, it is likely that mussels can cope with these exposures. However, because the overall IBR results suggest that a maximum response could be reached at <5 µg/L and this gains in environmental relevance, further lower realistic exposures deserve investigation.

## CONCLUSIONS

In the present study, the effects of Y exposure on the biochemical and physiological performance of the mussel *M. galloprovincialis* were described for the first time, demonstrating their ability to successfully deal with this rare‐earth element. Overall, organisms were most affected at the lowest concentration, when ETS, CAT, GR, CbEs, and GSTs activities were reduced, forcing organisms to use their energy reserves (GLY) and increase SOD activity to avoid cellular damage. Most of the inhibitions observed may have been a consequence of metabolic depression, with a shift to anaerobiosis, possibly due to valve closure. However, because at higher Y concentrations, the biomarker responses were less evident and the uptake rate of Y was lowered, the use of physiological adaptive processes not evaluated in the present study could be speculated. The long‐term risks of Y presence in coastal systems cannot be ignored because the levels that caused more significant biochemical alterations were those of environmental relevance.

## Supporting Information

The Supporting Information is available on the Wiley Online Library at https:/10.1002/etc.5508.

## Disclaimer

The authors declare that they have no known competing financial interests or personal relationships that could have appeared to influence the work reported in the present study.

## Author Contributions Statement

**Madalena Andrade**: Writing–original draft; Investigation; Formal analysis. **Amadeu M. V. M. Soares**: Funding acquisition. **Montserrat Solé**: Supervision; Writing—review & editing. **Eduarda Pereira, Rosa Freitas**: Conceptualization; Supervision; Funding acquisition; Writing—review & editing.

## Supporting information

This article contains online‐only Supporting Information.

Supplementary material 1.

Supplementary material 2.

## Data Availability

Data, associated metadata, and calculation tools are available from the corresponding author (rosafreitas@ua.pt). The data were restricted on request because they belong to a thesis of a PhD student not yet defended.
