# Supplementary material for: Threats of Pollutants Derived from Electronic Waste to Marine Bivalves: The Case of the Rare‐Earth Element Yttrium
Source: Environ Toxicol Chem. 2022 Dec 13;42(1):166–77. doi: 10.1002/etc.5508 (PMC10107937; doi:10.1002/etc.5508)
Supplement: Supplementary file 1 — Supplementary material 1. [file ETC-42-166-s002.docx]

Table 1 SM. Statistical results (PERMANOVA main test) performed to test the H_0_: no significant differences exists between mussels exposed to different concentrations of Y (0, 5, 10, 20 and 40 μg/L). Results comprise the degrees of freedom, *pseudo*-F and *p*-values, obtained for Y concentration in mussel’s soft tissue and physiological and biochemical parameters in *Mytilus galloprovincialis*. Significant values (*p* < 0.05) are in bold. BCF: Bioconcentration Factor; ETS: Electron Transport System; GLY: Glycogen; PROT: Protein; SOD: Superoxide dismutase; CAT: Catalase; GR: Glutathione Reductase; GSTs: Glutathione S-Transferases; LPO: Lipid Peroxidation; PC: Protein Carbonylation; CbEs: Carboxylesterases; AChE: Acetylcholinesterase.

|  | ***Degrees of freedom (df)*** | ***Pseudo-F value*** | ***p-value (MC)*** |
| --- | --- | --- | --- |
| Y concentration in tissue | 4 | 44.49 | **0.0001** |
| BCF | 3 | 7.793 | **0.0088** |
| ETS | 4 | 13.079 | **0.0008** |
| GLY | 4 | 11.285 | **0.0013** |
| PROT | 4 | 1.3976 | 0.2981 |
| SOD | 4 | 10.966 | **0.0008** |
| CAT | 4 | 5.9293 | **0.0078** |
| GR | 4 | 17.626 | **0.0002** |
| GSTs | 4 | 83.043 | **0.0001** |
| CbEs | 4 | 3.7604 | **0.0414** |
| LPO | 4 | 9.7633 | **0.0014** |
| PC | 4 | 8.3711 | **0.0029** |
| AChE | 4 | 7.9477 | **0.0041** |
