# Supplementary material for: Threats of Pollutants Derived from Electronic Waste to Marine Bivalves: The Case of the Rare‐Earth Element Yttrium
Source: Environ Toxicol Chem. 2022 Dec 13;42(1):166–77. doi: 10.1002/etc.5508 (PMC10107937; doi:10.1002/etc.5508)
Supplement: Supplementary file 2 — Supplementary material 2. [file ETC-42-166-s001.docx]

Table 2 SM. Pairwise comparisons (*p*-values (MC)) performed to test the H_0_: no significant differences exists between mussels exposed to different concentrations of Y (0, 5, 10, 20 and 40 μg/L). Comparisons between all treatments were performed for Y concentration in mussel’s soft tissue and the physiological and biochemical parameters in which the main test was significant. MHW: Significant values (*p* < 0.05) are in bold. BCF: Bioconcentration Factor; ETS: Electron Transport System; GLY: Glycogen; SOD: Superoxide dismutase; CAT: Catalase; GR: Glutathione Reductase; GSTs: Glutathione S-Transferases; LPO: Lipid Peroxidation; PC: Protein Carbonylation; CbEs: Carboxylesterases; AChE: Acetylcholinesterase.

| ***Comparisons*** | ***Y concentration in tissue*** | ***BCF*** | ***ETS*** | ***GLY*** | ***SOD*** | ***CAT*** | ***GR*** | ***GSTs*** | ***CbEs*** | ***LPO*** | ***PC*** | ***AChE*** |
| --- | --- | --- | --- | --- | --- | --- | --- | --- | --- | --- | --- | --- |
| 0 μg/L *vs* 5 μg/L | **0.0081** | - | **0.0059** | **0.0011** | **0.0098** | 0.0983 | **0.0036** | **0.0001** | **0.0153** | **0.0071** | 0.0872 | 0.2789 |
| 0 μg/L *vs* 10 μg/L | **0.0012** | - | 0.3271 | **0.0461** | **0.0055** | 0.3489 | **0.0041** | 0.9624 | 0.051 | 0.1059 | 0.0906 | 0.2359 |
| 0 μg/L *vs* 20 μg/L | **0.0004** | - | 0.0893 | **0.0127** | 0.2517 | 0.3418 | **0.006** | 0.6347 | 0.3653 | **0.0166** | 0.7031 | **0.0303** |
| 0 μg/L *vs* 40 μg/L | **0.0001** | - | 0.2147 | 0.1155 | 0.1111 | 0.2728 | 0.1209 | **0.003** | **0.0145** | 0.0547 | 0.1302 | 0.0708 |
| 5 μg/L *vs* 10 μg/L | 0.1091 | 0.5811 | **0.002** | **0.014** | 0.9025 | 0.1953 | 0.1304 | **0.0001** | 0.0676 | **0.0051** | **0.0051** | 0.1286 |
| 5 μg/L *vs* 20 μg/L | **0.0425** | 0.0643 | **0.0019** | 0.0862 | **0.0225** | **0.005** | 0.6513 | **0.0006** | 0.1564 | 0.9336 | **0.0246** | 0.0700 |
| 5 μg/L *vs* 40 μg/L | **0.0012** | **0.0254** | **0.0039** | **0.0097** | **0.0401** | **0.0128** | **0.0079** | **0.0002** | 0.1167 | 0.9369 | **0.0004** | **0.0486** |
| 10 μg/L *vs* 20 μg/L | 0.3917 | **0.0342** | 0.1511 | 0.2231 | **0.0168** | **0.0279** | 0.2619 | 0.6669 | 0.8216 | **0.011** | 0.094 | **0.0265** |
| 10 μg/L *vs* 40 μg/L | **0.0051** | **0.0054** | 0.5788 | 0.5132 | **0.023** | **0.0433** | **0.0072** | **0.005** | 0.3754 | **0.0234** | 0.3300 | 0.6173 |
| 20 μg/L *vs* 40 μg/L | **0.0093** | 0.0912 | 0.4208 | 0.1082 | 0.5661 | 0.6395 | **0.014** | **0.0472** | 0.559 | 0.896 | 0.1303 | **0.0154** |
